# Koch’s Treatment—and Other Treatments

**Published:** 1891-07

**Authors:** 


### KOCH'S TREATMENT—AND OTHER TREATMENTS.

We may say regretfully, that the specific treatment of tuberculosis, as devised by Prof. Koch, is no more. It is now enjoying a condition of innocuous desuetude that is almost oppressive. It is departed, and the places which once knew it now know it no more. Yes, it has gone to meet the gas treatment of Bergeon, and in the land of the Great Departed, among the shades of other deceased medical failures and superstitions, it has doubtless found a congenial companionship in the tar-water of Bishop Berkeley, and the metallic tractors of Perkins.

We believe we are safe in saying that no other therapeutic suggestion or discovery (*sic*) was ever received more confidently and with greater enthusiasm. Prof. Koch states incidentally in August that he has been experimenting upon guinea pigs with a certain substance which conferred upon them immunity to inoculations of the tubercle bacillus and which was capable of arresting the tuberculous process in those already the subjects of the disease. Such an announcement could not fail to excite interest and to create hope. The public mind, both lay and medical, became more and more impatient, until finally under official—almost military—duress, the discoverer was forced to issue to the world his famous letter of November 14th. What happened then is well-known. There followed the most remarkable pilgrimage of doctors which has been seen in the history of medicine. Two thousand physicians, and probably as many patients, immediately flock to the German capital, all eager to possess the new therapeutic talisman. Poetically,

> From the four corners of the earth they come,
> To kiss this shrine, this mortal, breathing saint;
> * * * * * *
> The watery kingdom, whose ambitious head
> Spits in the face of heaven, is no bar
> To stop the foreign spirits, but they come,
> As o'er a brook, to learn of Kochine.

Enthusiasm ran high, and it did seem as if this greatest enemy of the human family had met a capable antagonistic at last. We quote the words of Nothnagel: "We face one of the greatest intellectural achievements in the province of medicine for centuries past. The discovery has a far wider scope than Jenner's, and is, perhaps, the greatest feat in science. . . . . . The present moment is among the most sublime that humanity has known." So far as the writer knows Prof. Nothnagel has failed to enlighten us on his present views as to this greatest feat in science.

During the past eight months Koch's proposed remedy has received a careful and painstaking trial the world over, under the most favorable circumstances. No other therapeutic agen was ever proposed which met with a more rapid, more widespread, and more thorough investigation. Cases after cases of remarkable cures of the different tuberculous conditions were quickly reported, embracing all degrees of severity. Hospital physicians rushed into print with their "notes" and "observations." It soon became evident, however, that many patients did not improve, that others were not suitable for this treatment, that others were actually made worse by it. Finally (about April 1st), an official report was issued by the German government which contained some cold arithmetic for the advocates of the tuberculin treatment. Out of 951 cases of phthisis only 13 were reported as cured, 175 markedly improved, 194 slightly improved, 586 not improved and 46 died. Under our former methods of treatment, which were represented by climate, diet and supportive medication, we were able to do better than this. Loomis (*Practical Medicine*) says that one-sixth of his recorded cases (or 16⅔ per cent.) during the last ten years have recovered. Out of 640 cases observed by Flint during a period of thirty-four years, recovery took place in 44, or 6.8 per cent. (*Pepper's System of Medicine.*) Against these percentages stands the 1.3 per cent. of the new method.

It may be, however, that the occupation of tuberculin is not entirely gone. There seems to be a field of usefulness for it as an adjunct to other methods of treatment. It may yet be made to accomplish much good when used in connection with favorable hygienic and climatic surroundings. Jacobi believes that the future successful treatment of pulmonary consumption will consist of a combination of climatic cures with a careful and persistent use of tuberculin (*Med. Rec.*, *Mar.* 7). Such, it appears, is the new method's only hope.

The life history of tuberculin is thus put in a nut-shell by the witty editor of the *Medical Press:*

Act I.—*Eureka.*
Act II.—*Vici.*
Act III.—*Ave, morituri te salutant.*
Act IV.—*De mortuis nil nisi bonum.*
Epitaph—*Fuit.*

To which we will add—*Non omne, quod nitet, aurum est.*

Concerning the other plans of treatment which have recently been devised and practiced, we need not say very much. These may be enumerated as follows: 1. The intra-venous or subcutaneous injection of dog's or goat's serum. This is the method first practiced by Messrs. Herricourt and Richet. 2. The treatment by solutions of iodine and chloride of gold and sodium. Drs. Shurly and Gibbes are responsible for this system. 3. The cantharidinate of potash, introduced by the enterprising Prof. Liebreich. 4. The compressed air treatment of Prof. Germain Sée. Here phthisical patients pass several hours daily in a close chamber subjected to a pressure of an atmosphere and a half. The compressed air is saturated with fumigations of creasote and eucalyptus.

And even these are not all. It has been said of all these plans successively, in a general way, thus: The experiments which have been conducted by Prof. ———, with his new treatment for tuberculosis, have given most encouraging results. Patients in the early stages of the disease show rapid and marked improvement; the distressing symptoms are all relieved and the general condition of the patient is greatly ameliorated. These generalizations, however, do not demonstrate. We are skeptical, but open to conviction. In the meantime we submit the following from Byron:

> Thus saith the preacher: "Nought beneath the sun
> Is new": yet still from change to change we run;
> What varied wonders tempt us as they pass!
> The cow-pox, tractors, galvanism and gas,
> In turns appear, to make the vulgar stare,
> Till the swollen bubble bursts—*and all is air!*

